# Nucleolar Localization of RNA Binding Proteins Induced by Actinomycin D and Heat Shock in *Trypanosoma cruzi*

**DOI:** 10.1371/journal.pone.0019920

**Published:** 2011-05-24

**Authors:** Ezequiel Názer, Ramiro E. Verdún, Daniel O. Sánchez

**Affiliations:** 1 Instituto de Investigaciones Biotecnólogicas-Instituto Tecnológico Chascomús, UNSAM-CONICET, San Martín, Provincia de Buenos Aires, Argentina; 2 Leonard M. Miller School of Medicine, University of Miami, Miami, Florida, United States of America; Federal University of São Paulo, Brazil

## Abstract

In this work we show that under Actinomycin D (ActD) treatment, several RNA Binding Proteins (RBPs) involved in mRNA metabolism are relocalized into the nucleolus in *Trypanosoma cruzi* as a specific stress response. ATP depletion as well as kinase inhibition markedly reduced the nucleolar localization response, suggesting that an energy-dependent transport modulated by the phosphorylation status of the parasite might be required. Deletion analyses in one of such proteins, TcSR62, showed that a domain bearing basic amino acids located in the COOH terminal region was sufficient to promote its nucleolar relocalization. Interestingly, we showed that in addition to RBPs, poly(A)+ RNA is also accumulated into the nucleolus in response to ActD treatment. Finally, we found out that nucleolar relocalization of RBPs is also triggered by severe heat shock in a reversible way. Together, these results suggest that the nucleolus of an early divergent eukaryote is either able to sequester key factors related to mRNA metabolism in response to transcriptional stress or behaves as a RBP processing center, arguing in favour to the hypothesis that the non-traditional features of the nucleolus could be acquired early during evolution.

## Introduction

*Leishmania spp, Trypanosoma cruzi and Trypanosoma brucei* are single-celled parasitic protozoa that, together, cause millions of deaths in developing countries [1], [2]. Since these parasites have a complex life cycle, alternating between an insect vector (blood-sucking bugs) and mammalian hosts, they are exposed to continuous and sudden environmental changes during their transmission. As a consequence, they need to reprogram the expression of many proteins, as fast as possible, to deal with completely different environmental conditions. The adaptation process involves large changes both in their metabolism [3] and in their morphology [3], which are driven by particular gene expression mechanisms [4]. Unlike higher eukaryotes, trypanosomatids do not regulate gene expression at the level of transcription initiation [4], [5]. Instead, in these organisms, the main control point has been shifted to the post-transcriptional level [4].

In recent years, it has also been shown that stress granules (SGs) and processing bodies (PBs) are important players in the post-transcriptional regulation of gene expression in both yeast and mammalian cells [6]. SGs and PBs are spatially, compositionally, and functionally linked places, where mRNAs are sorted, stored, degraded and remodelled [7], controlling in this way mRNA translation/decay, particularly during stress conditions. In trypanosomes, the presence of cytoplasmic granules that are induced by different stress conditions has recently been shown [8]–[10]. In *T. brucei,* SGs and PBs induced by severe heat shock have a composition similar to those present in mammals [10].

More recently, the nucleolus has also been implicated in a variety of cellular processes apart from the well-known rRNA transcription and ribosome assembly. Some of these additional functions are related to the regulation of mitosis, cell-cycle progression, biogenesis of ribonucleoprotein particles and stress response to a variety of stressors [11]. With regards to the role of the nucleolus in the stress response, it has been suggested that the nucleolus might have a double function during stress: it may act as a sensor [12] and as a coordinator of the cellular response [13].

Several nucleolar proteomic analyses also suggest a broad participation of the nucleolus in different cellular processes [14]–[17]. Regarding RNA metabolism, the presence of several transcription factors, splicing factors and different RNA Binding Proteins (RBPs), such as SR proteins and heterogeneous nuclear ribonucleoproteins (hnRNPs), suggests the participation of the nucleolus in many RNA processes such as transcription, pre-mRNA processing, degradation, transport and localization. Comparison of human and yeast nucleolar proteomes have shown that there are many homologous proteins among them that support the notion that the nucleolar proteome and, therefore, its additional functions, might have been conserved during evolution [16].

In Trypanosomes, an early divergent eukaryote group, the nucleolus presents some important differences compared to human and yeast. For example, at the structural level, the fibrillar centres seem to be absent [18], [19], whereas at the functional level, the rRNA processing is quite different from that observed in most eukaryotes regarding both the processing itself and the mature rRNA molecules generated [20]. Another interesting feature is that nucleolar structures in *T. cruzi* infective parasite forms are dispersed in the nucleoplasm, suggesting that the nucleolar architecture might be reorganised during this particular life-cycle stage [21]. In addition, it has also been reported that the nucleolus disperses when a culture of epimastigote cells reach the stationary phase [22]. Taking all these data into consideration, Kelly and collaborators speculated that the nucleolus could also participate as a stress sensor in trypanosomes, being dispersed under particular stress conditions which could finally promote differentiation [22].

In this work, we provide some evidences that suggest that the nucleolus of *T. cruzi* is indeed involved in the parasite stress response. We show that a subset of RBPs involved in mRNA metabolism is accumulated in the nucleolus as a specific stress response triggered by Actinomycin D (ActD) treatment. ATP depletion as well as kinase inhibition markedly reduced the nucleolar localization response, thus suggesting that an energy-dependent transport modulated by the phosphorylation status of the parasite might be required.

In addition to RBPs, we showed that poly(A)+ RNA is also accumulated into the nucleolus in response to ActD. Finally, we observed that nucleolar accumulation of RBPs is also triggered by severe heat shock in a reversible way --a stress that also induces a transcriptional shut down--, suggesting that RBPs nucleolar relocalization may be part of a physiological stress response.

## Results

### The RBP TcSR62 from *T. cruzi* is relocalized from nuclear speckles to the nucleolus upon ActD treatment

**Figure 1:**
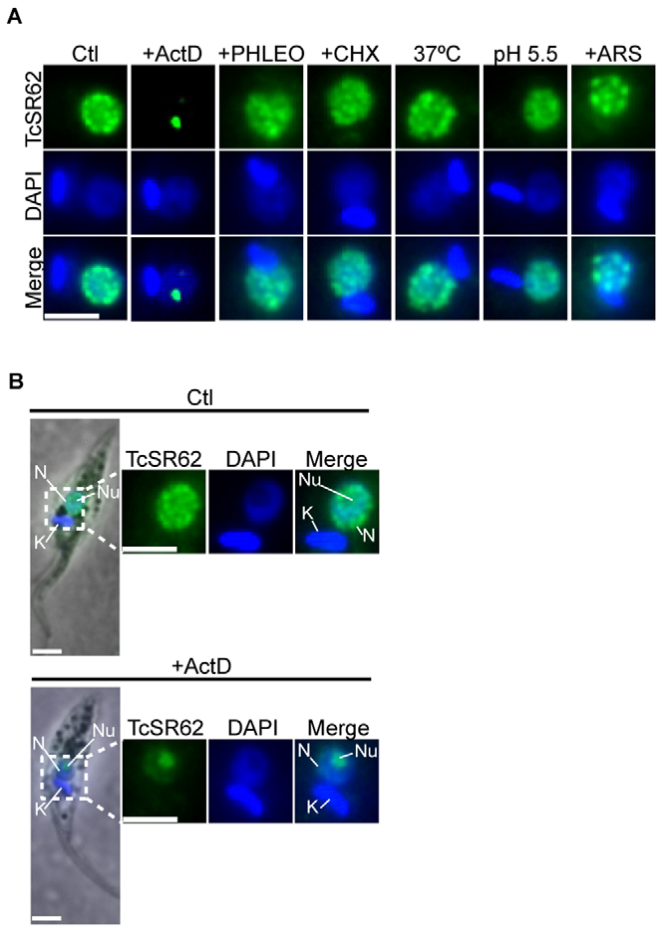
TcSR62 behaviour under several stress conditions. (A) Transcription inhibition was induced incubating epimastigotes with ActD for 24 h. Genotoxic stress was induced with phleomycin (phleo), and protein synthesis inhibition with cycloheximide (CHX) for 24 h, respectively. Oxidative stress was induced subjecting the cells to sodium arsenite (ARS) for 2 h. Acid pH stress was induced incubating cells in BHT pH 5.5 for 24 h. Heat shock was induced after incubating cells at 37°C for 24 h. (B) Localization detail of TcSR62 in parasites subjected or not to transcription inhibition. TcSR62 (green) was detected by immunofluorescence. Nuclei were counterstained with DAPI (blue). N: nucleus, K: kinetoplast, Nu: nucleolus. Size bars represent 2 µm. Representative nuclei are shown.

**Figure 2:**
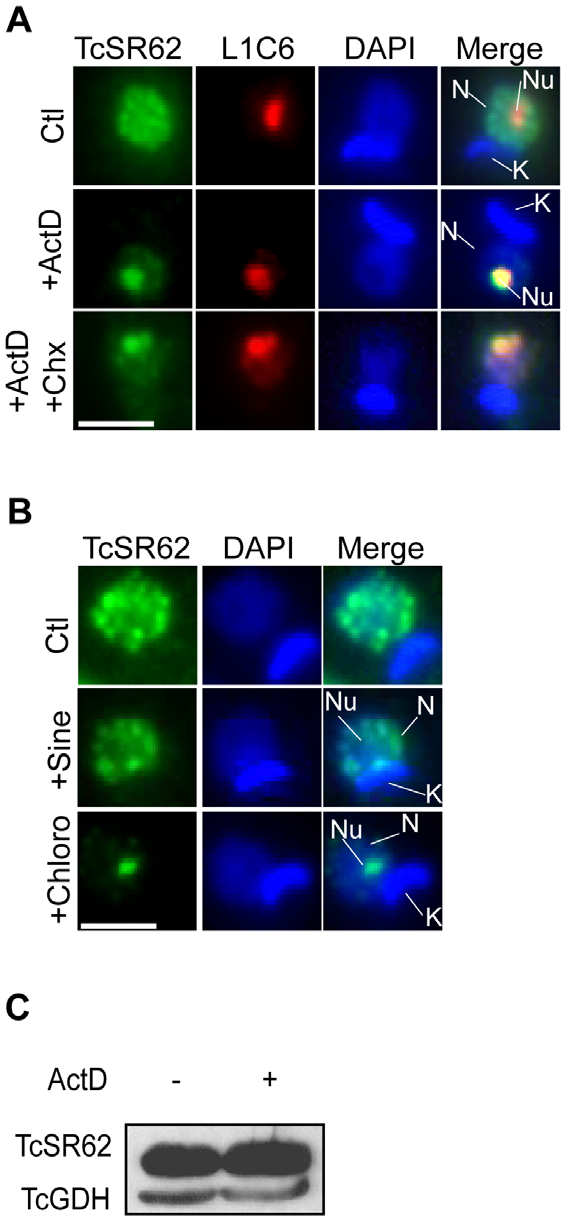
TcSR62 is relocalized to the nucleolus after ActD treatment. (A) Immunofluorescence images of double staining for TcSR62 (green) and the nucleolar marker L1C6 (red) in ActD-treated and untreated epimastigotes (top and middle panels) or in ActD-Chx-treated epimastigotes (bottom panels). The fourth column on the right is an overlap of the TcSR62, L1C6 and DNA stain, showing nucleolar relocalization of TcSR62 upon transcription inhibition. Green and red pixels overlapped in the digital images yielded yellow signals. (B) Immunofluorescence images of double staining for TcSR62 (green) and DAPI in untreated (top panels), sinefungin-treated epimastigotes (middle panels) or in chloroquine-treated parasites (bottom panels). The third column on the right is an overlap of the TcSR62 and DNA stain. Nuclei were counterstained with DAPI (blue). N: nucleus, K: kinetoplast, Nu: nucleolus. Size bars represent 2 µm. Representative nuclei are shown. (C) Immunoblot showing an extract of 3×10^7^ epimastigotes under normal growth conditions or exposed to transcription inhibition. The membrane was sequentially stained with TcSR62 and TcGDH antibodies, respectively.

TcSR62 (Tc00.1047053511621.50) is a RBP belonging to the SR-related protein family. SR and SR-related proteins are implicated in several functions related to mRNA metabolism [23], [24]. Work under way in our lab. suggests that TcSR62 is involved in mRNA processing/stability since its over-expression in *T. brucei* affects the trans-splicing process and decrease the abundance of several mRNAs (Mendiondo et al., in preparation). To further characterize this protein, we analyzed its distribution under different stress conditions by subjecting parasites to a broad spectrum of metabolic and environmental conditions. As shown in Figure 1A, its distribution remained mostly unchanged compared to untreated parasites. However, following 24 h of transcription inhibition induced by ActD, TcSR62 underwent a striking shift strongly concentrating in a bright globular structure that resembled the nucleolus as it colocalized with a weaker area of staining with DNA-specific dye DAPI (Figure 1A, +ActD panels). Similar results were obtained when parasites were treated with chloroquine (Figure 2B, bottom panels), another transcription inhibitor which has been used previously in *T. brucei* [25]. Then, a time-course experiment was carried out to know the timing of this response (Figure S1). After 3–9 h of ActD treatment, TcSR62 distribution started to change from several uniformly stained speckles to few larger and brighter ones. At later times (9–24 h), TcSR62 was also present in a large dot (most likely the nucleolus, see below), being more noticeable at 24 h in almost the whole parasite population (86% of parasites). Taking this last result into account, further experiments involving ActD were analyzed at 24 h.

We then wished to confirm the nucleolar localization of TcSR62 after transcription inhibition by performing colocalization studies with Mab L1C6, a monoclonal antibody that recognizes an antigen localized in the nucleolus of *T. brucei* [26], [27], which also cross-reacts with a nucleolar antigen in *T. cruzi* [22], [28]. After subjecting parasites to ActD, the antigen recognized by L1C6 was relocated from the nucleolus to the nucleoplasm in 70% of the cells. However, the remaining 30% of the cells exhibiting L1C6 nucleolar localization allowed us to colocalize it with TcSR62 to the nucleolus (Figure 2A, middle panels). To know whether TcSR62 might be accumulating into the nucleolus as a consequence of *de novo* protein synthesis, we treated parasites with both ActD and the translation inhibitor cycloheximide. Nucleolar accumulation of TcSR62 could also take place under this condition (Figure 2A, bottom panels), suggesting that *de novo* protein synthesis could not account for its nucleolar localization upon ActD treatment. Importantly, the decrease observed in the labelling intensity in speckles was not due to TcSR62 degradation, as the total amount of this protein remained constant in the parasite as seen in the immunoblot shown in Figure 2C.

These results show that the RBP TcSR62 is specifically relocalized to the nucleolus in response to a particular stress condition, suggesting a possible role of the nucleolus in the *T. cruzi* stress response.

### Trans-splicing inhibition does not promote TcSR62 nucleolar relocalization

Since transcription and trans-splicing are two closely linked processes in trypanosome mRNA maturation and taking into account that TcSR62 is involved in mRNA processing (our unpublished results), we wondered whether TcSR62 nucleolar relocalization might be either a direct response promoted by ActD or a side effect generated by a trans-splicing decrease. Therefore, we examined whether cellular stress induced by trans-splicing inhibition could have any effect on TcSR62 localization. Figure 2B (middle panels) shows the cellular localization pattern of TcSR62 in parasites exposed to sinefungin, an antibiotic which inhibits trans-splicing in trypanosomes [29], [30]. Following 24 h of sinefungin treatment, TcSR62 did not show nucleolar localization, suggesting that such response is not directly mediated by trans-splicing inhibition.

### ActD treatment impacts on other RNA Binding Proteins

**Figure 3:**
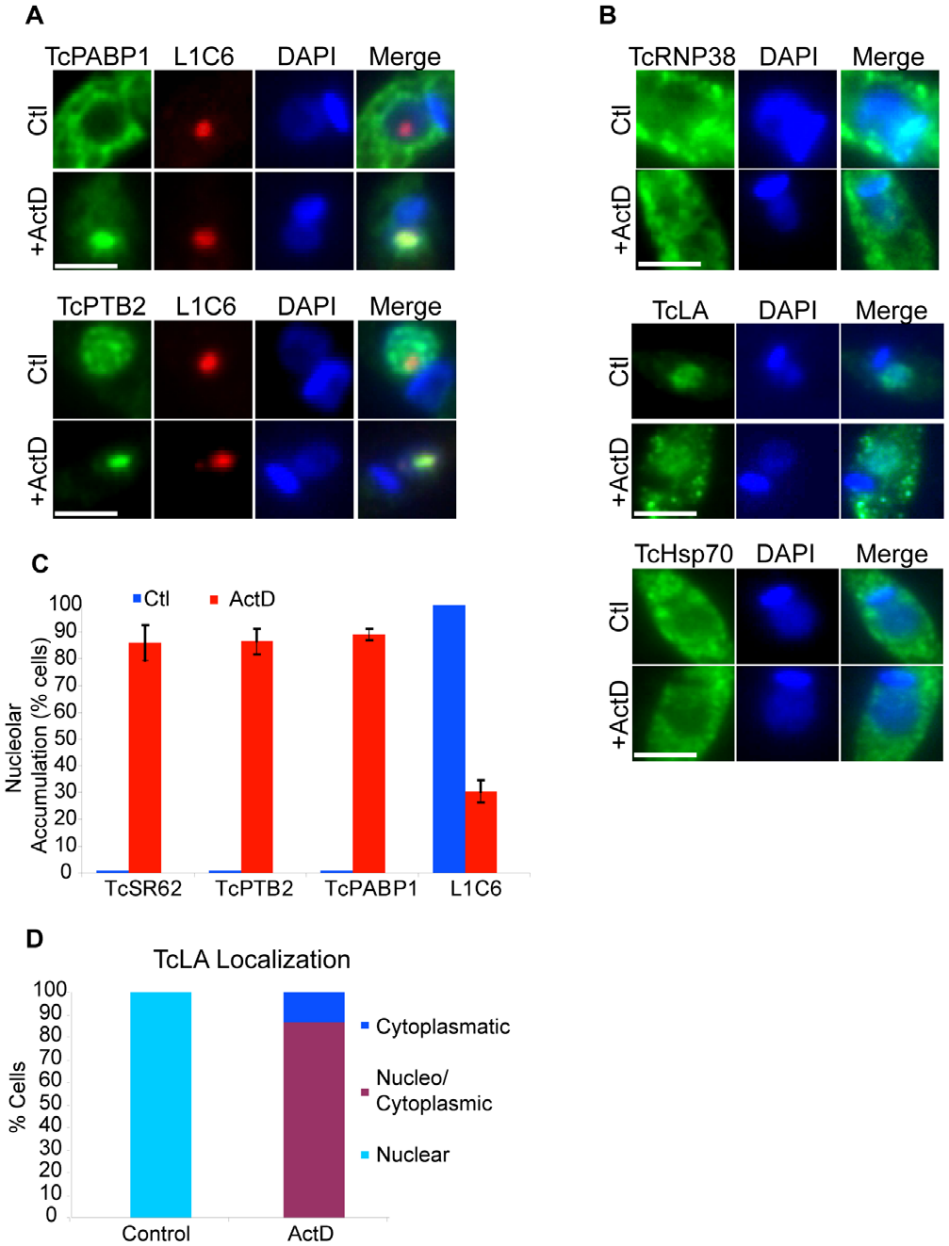
Effects of ActD treatment on the localization of several RNA binding proteins and TcHSP70. (A) Immunofluorescence images of TcPTB2, TcPABP1 in ActD-treated and untreated epimastigotes. Each protein (in green) was colocalized with the nucleolar marker L1C6 (red). Nuclei were counterstained with DAPI (blue). The fourth column on the right is an overlap of each protein, L1C6 and DNA staining. Green and red pixels overlapped in the digital images yielded yellow signals. (B) Immunofluorescence images for TcRNP38, TcLA and TcHSP70. Each protein is shown in green. Nuclei were counterstained with DAPI (blue). The third column on the right is an overlap of each protein and DNA stain. Size bars represent 2 µm. Representative nuclei are shown. The graphic in panel (C) shows the percentage of cells showing nucleolar localization for TcSR62, TcPTB2, TcPABP1 and L1C6 in control (blue bars) and Act-D-treated cells (red bars). (D) Quantitative analysis of TcLA behaviour under transcription inhibition treatment. The results are expressed as mean +/− SD from at least three independent experiments.

Since it has been previously shown that RNA Pol II is dispersed through the nucleoplasm in response to ActD treatment in *T. cruzi* [31], and that TcSR62 is accumulated into the nucleolus in response to the same treatment (see above), we wondered whether nucleolar localization of TcSR62 was either a specific behaviour of such protein or could also be extended to other RBPs. To explore this, we first examined the effect of ActD treatment on the subcellular localization of several RBPs known to be involved in RNA metabolism such as TcPABP1, TcPTB2 and TcLA. TcPABP1 is a Poly(A) Binding Protein (PABP) involved in the stabilization of polyadenylated mRNAs and the interaction of the poly(A) tail with the translation initiation complex. In *T. cruzi*, the PABP1 homologue is a 66 kDa protein constitutively expressed in all stages of the parasite [32], [33]. TcPTB2, also known in *T. cruzi* as DRBD4 [32], and whose *T. brucei* orthologue, TbPTB2, was described by Michaeli's group [34], is a homologue of the Polypyrimidine Tract Binding protein (PTB). TcLA is a multifunctional RBP which is involved in splice leader maturation and tRNA intron removal in *T. brucei* [35]. Under ActD treatment, TcPABP1 and TcPTB2 were relocalized into the nucleolus in most cells, as shown by colocalization with the L1C6 nucleolar marker (Figure 3A and S4A). TcLA, on the other hand, was not accumulated in the nucleolus, but partially mobilized to the cytoplasm where it localized mainly to the cell periphery with a dotted pattern (Figure 3B); in addition, about 13% of the cells showed exclusively a cytoplasmic localization (not shown). To further extend our analysis we also evaluated two RBPs which have recently been characterized in trypanosomes: TcSF3b155, a splicing factor, and TcFIP1, a protein involved in the polyadenylation reaction of mRNAs. Both proteins have been reported to localize in nuclear speckles [36], [37]. As expected, both proteins displayed a nuclear speckled pattern in untreated parasites; however, after exposing the parasites to ActD, these two proteins were relocalized to the nucleolus (Figure S2A and B). We then evaluated two proteins unrelated to RNA processing: TcRNP38, a cytoplasmic RBP characterized by bearing only one RRM domain and whose *T. brucei* orthologue does not show phenotypic alterations after its expression is knocked down [38]; and TcHSP70, a stress response protein that mobilizes from the cytoplasm to the nucleus in response to heat shock [39], and whose mammalian counterpart also relocates into the nucleolus under the same stress [40]–[42]. As shown in Figure 3B, S4B and S4C, neither TcRNP38 nor TcHSP70 were relocalized to the nucleolus under ActD treatment. Figure 3C shows a quantification of the experiments with TcSR62, TcPTB2, TcPABP1 and L1C6, whereas Figure 3D shows a quantification of the experiment with TcLA.

These results suggest that ActD treatment in this parasite induces a specific response involving a particular subset of RBPs, which are related to mRNA metabolism. Interestingly, nuclear (TcSR62, TcPTB2, TcSF3b155 and TcFIP1) as well as cytoplasmic (TcPABP1) RBPs were relocalized to the nucleolus.

### Nucleolar accumulation of RNA Binding Proteins under ActD treatment depends on an active transport mechanism

**Figure 4:**
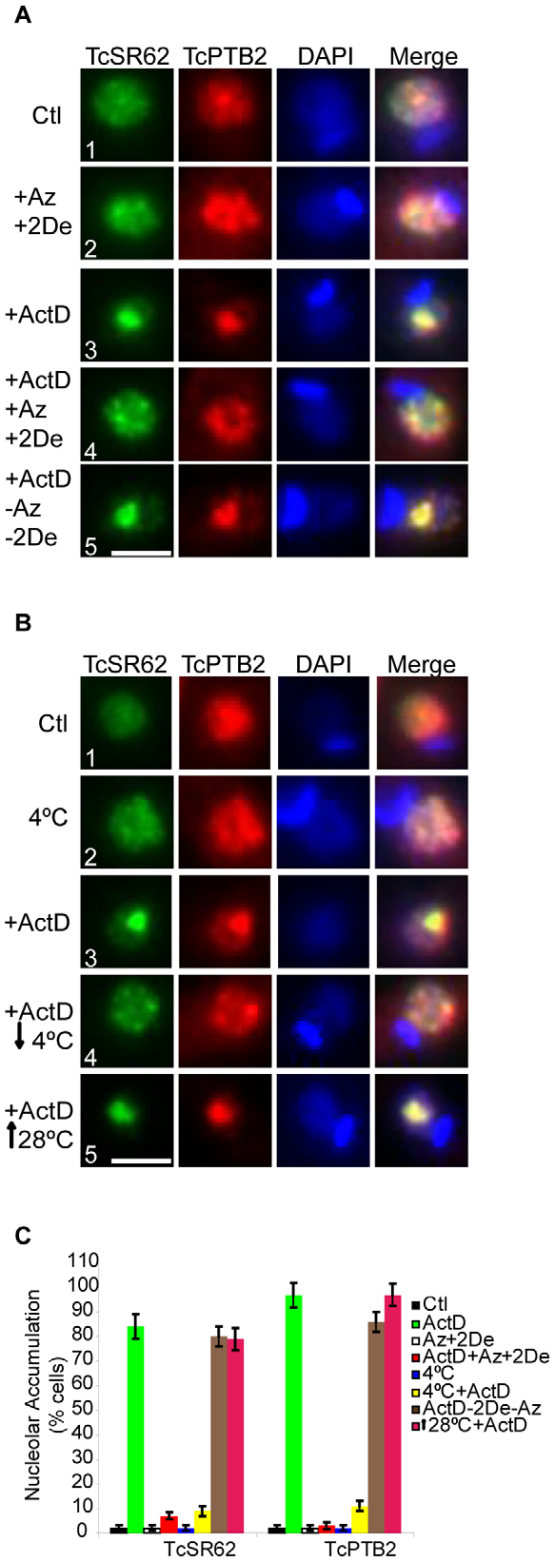
Nucleolar accumulation of RBPs upon ActD treatment depends on an active transport mechanism. (A) Epimastigotes were incubated with both sodium Azide (Az) and 2-Deoxy-Glucose (2De) and ActD at 28°C for 24 h. (B) Epimastigotes were incubated with ActD at 4°C for 24 h. Then, immunofluorescence studies were performed using antibodies against TcSR62 (green) and TcPTB2 (red). Recovery of both treatments was allowed either by washing up the cultures and then incubating with fresh medium (−Az−2De+ActD) or reincubating at 28°C (↑28°+ActD) for 24 h. Each inhibitor treatment alone or cell culture at 4°C, are shown as controls. Nuclei were counterstained with DAPI (blue). The fourth column on the right is an overlap of the TcSR62 and TcPTB2 and DNA stain. Green and red pixels overlapped in the digital images yielded yellow signals. Size bars represent 2 µm. Representative nuclei are shown. The graphic in panel (C) is a quantification of the experiments shown in panels (A) and (B). Results are expressed as mean +/− SD from at least three independent experiments.

The observation of nucleolar migration following ActD treatment led us to examine whether this response depends on an active nucleolar transport, or passive diffusion towards the nucleolus and retention in this organelle by binding to a nucleolar component. To distinguish between these two possibilities, we took advantage of two metabolic inhibitors, namely 2-deoxy-D-glucose (2De) and sodium Azide (Az) for ATP depletion. The former inhibits glycolysis, whereas the latter inhibits the production of ATP by oxidative phosphorylation [43]. Previous works have demonstrated that these two metabolic inhibitors promote nuclear transport inhibition in HeLa cells [43], yeast [44], and also in *T. brucei* [45]. We evaluated the behaviour of TcSR62 and TcPTB2 upon ATP depletion in untreated and ActD-treated epimastigote cells (Figure 4A). Neither protein changed its subnuclear localization pattern after treating parasites simultaneously with both metabolic inhibitors for 24 h (Figure 4A, compare panels 1 and 2). However, nucleolar relocalization of both proteins, promoted by ActD, was almost completely abolished in the presence of these metabolic inhibitors (Figure 4A, compare panels 3 and 4). Finally, to know whether the nucleolar transport inhibition was reversible, we first treated parasites with ActD in the presence of both inhibitors for 24 h, washed them twice with PBS, refed them with fresh medium and then incubated them for another 24 h. As it can be seen in Figure 4A (panels 4 and 5), both proteins were relocalized to the nucleolus in the majority of the cells, suggesting that an active metabolism is essential for transporting these proteins from nuclear speckles to the nucleolus.

Active transport in trypanosomes can also be diminished by using low temperatures [45]. To test whether TcSR62 and TcPTB2 nucleolar localization could also be inhibited at low temperature, epimastigotes were incubated in the presence of ActD at 4°C for 24 h. Figure 4B shows that, under this condition, TcSR62 and TcPTB2 were detected in speckles and not into the nucleolus (Figure 4B, panels 3 and 4). However, nucleolar accumulation of both proteins was resumed in most cells after increasing the temperature from 4°C to 28°C for another 24 h (Figure 4B, panels 4 and 5). Figure 4C shows a quantification of the experiments shown in Figure 4A and 4B.

From these set of experiments, we concluded that nucleolar accumulation of RBPs in response to ActD is mediated by an energy-dependent and dynamic process.

### Nucleolar relocalization of RBPs induced by ActD is blocked by inhibiting phosphorylation but not dephosphorylation

**Figure 5:**
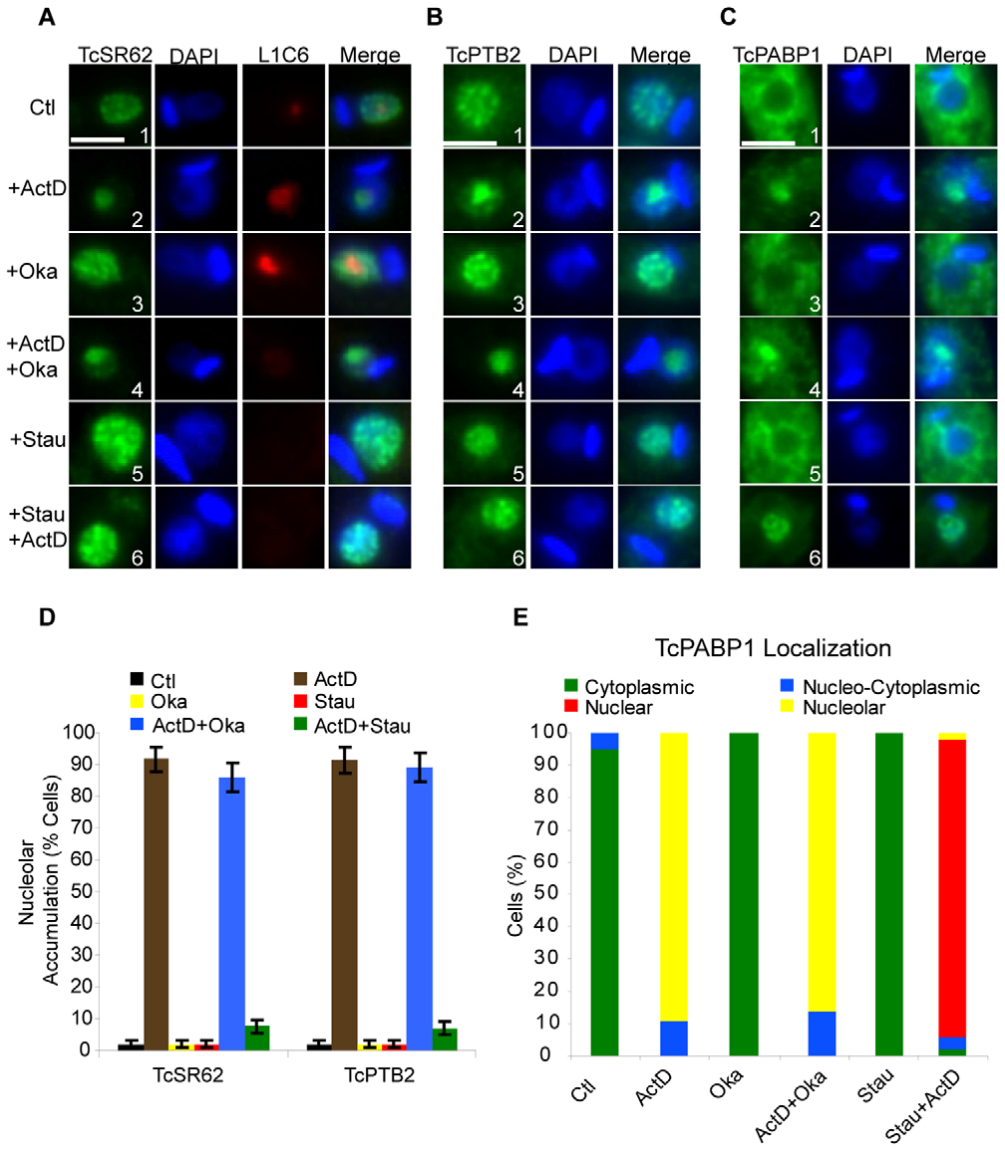
Stress-induced nucleolar localization of RBPs is inhibited by blocking phosphorylation but not dephosphorylation. Epimastigotes were incubated either with both okadaic acid (Oka) and ActD for 24 h or first preincubated with staurosporine (Stau) for 16 h and then with ActD for 24 h. Panel (A) shows TcSR62 (green), L1C6 (red) and DAPI. The fourth column on the right is an overlap of the TcSR62 and L1C6 and DNA stain. Green and red pixels overlapped in the digital images yielded yellow signals. (B) TcPTB2 (green), (C) TcPABP1 (green), each counterstained with DAPI. The third column on the right is an overlap of each protein and DNA stain. Each inhibitor treatment alone is shown as control. Nuclei were counterstained with DAPI (blue). Size bars represent 2 µm. Representative nuclei are shown. The graphic in panel (D) is a quantitative analysis of the experiments shown in panels (A) and (B). The graphic in panel (E) is a quantitative analysis of TcPABP1 behaviour. Results are expressed as mean +/− SD from at least three independent experiments.

It is well known that phosphorylation/dephosphorylation processes play an important role in protein localization as well as in the modulation of the interaction of proteins such as SF2/ASF (a well-studied SR protein), PTB and PABP [46]–[50]. To test the possibility that nucleolar relocalization of RBPs in *T. cruzi* might be also regulated by such mechanism, we investigated their behaviour in parasites subjected to ActD treatment in the presence of either okadaic acid (a specific inhibitor of protein serine/threonine phosphatases 1, 2A and 2B) [51] or staurosporine (a broad-spectrum protein kinase inhibitor) [52]. Although we are aware that these drugs affect the overall cellular phosphorylation/dephosphorylation cycle, they have been --and still are-- used extensively to investigate whether changes in the phosphorylation state of proteins are responsible for altering their activity and/or their dynamics within cells [53]–[56]. The results shown in Figure 5 demonstrate that neither okadaic acid (Figure 5, panel 3) nor staurosporine alone (Figure 5, panel 5) affected the speckled pattern of TcSR62 or TcPTB2 (Figure 5A and 5B, respectively) or the cytoplasmic localization of TcPABP1 (Figure 5C). However, ActD-induced nucleolar accumulation of these proteins was inhibited upon staurosporine but not okadaic acid treatment (see panels 6 and 4, respectively). Figure 5D shows a quantification of the experiments illustrated in Figure 5A and 5B. Interestingly, upon treatment of parasites with ActD and staurosporine, TcPABP1 was able to mobilize from the cytoplasm to the nucleus, accumulating throughout the nucleoplasm, but it was unable to accumulate into the nucleolus in most cells (see Figure 5E for a quantitative analysis). It should be pointed out that nucleolar accumulation inhibition of RBPs by staurosporine was effective only when the cells were pretreated with this drug 16 h before ActD was added and further incubated for 24 h. Simultaneous treatment with both drugs could not prevent nucleolar relocalization of the RBPs analyzed.

Regardless of whether this is a direct or an indirect effect of the inhibitor on the RBPs evaluated, these results suggest that ActD-induced nucleolar accumulation of RBPs probably involves a mechanism comprising the activity of protein kinases but not of okadaic acid-sensitive phosphatases. Interestingly, although ActD treatment triggers TcPABP1 nucleolar accumulation, its intranuclear transport from the nucleoplasm to the nucleolus seems to be dependent on the activity of a yet unknown kinase(s).

### Nucleolar targeting of TcSR62 is localized in the COOH terminal region

**Figure 6:**
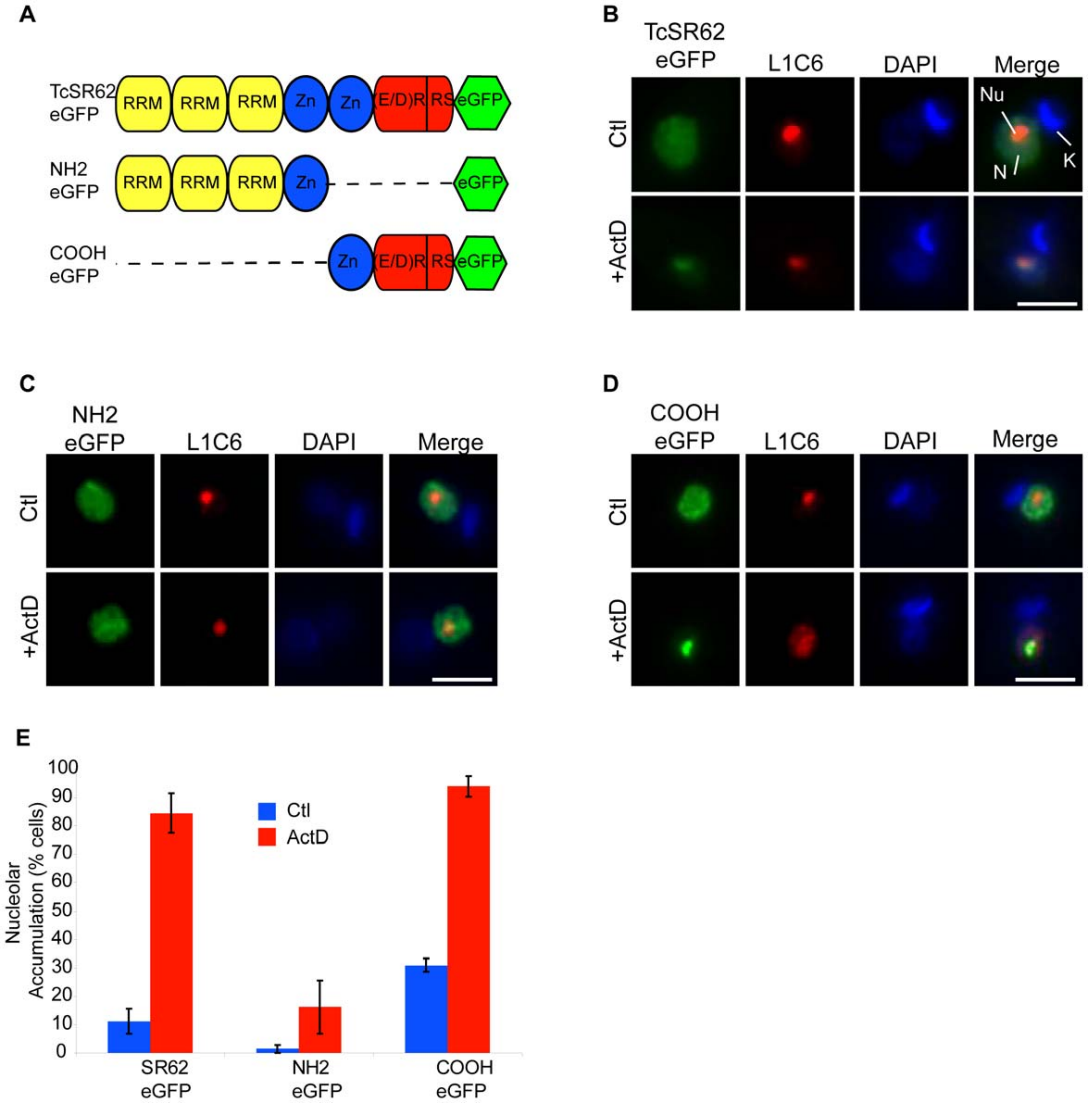
Nucleolar determinants of TcSR62 are located in the COOH terminal region. (A) Cartoon showing TcSR62 main domains and mutants expressed as eGFP fusion proteins in *T. cruzi*. (B) SR62-eGFP (C) NH_2_-eGFP (D) COOH-eGFP parasites were untreated or incubated with ActD for 24 h. Fusion proteins are shown in green and L1C6 in red. The fourth column on the right is an overlap of each fusion protein, L1C6 and DNA stain. Green and red pixels overlapped in the digital images yielded yellow signals. Nuclei were counterstained with DAPI (blue). N: nucleus, K: kinetoplast, Nu: nucleolus. Size bars represent 2 µm. Representative nuclei are shown. The graphic in panel (E) is a quantification of the experiments showed in panels (B), (C) and (D). Results are expressed as mean +/− SD from three independent experiments.

TcSR62 has a complex but well-defined modular domain structure containing three RRM domains located in its NH_2_-terminal and an Arg-rich COOH-terminal domain which contains two CCHC Zinc finger motifs and two partially overlapping subdomains, a large and ER/DR-rich one and a small and SR-rich one located right at the C-terminal end (Figure 6A). To determine which domain(s) of TcSR62 were required to allow its nucleolar localization, we generated three C-terminal eGFP fusion constructs (Figure 6A) containing the full-length TcSR62 gene (SR62-eGFP), the NH_2_ terminal region (NH_2_-eGFP) or the COOH terminal region (COOH-eGFP). Parasites transfected with each eGFP-tagged construct were analyzed either before or after ActD treatment.

Under normal conditions, the full-length TcSR62-eGFP protein was localized mainly to the nucleus in a typical diffused and speckled pattern (Figure 6B, top panels), in agreement with immunolocalization assays described before for the endogenous TcSR62 protein. On the other hand, we observed differences between both deletion constructs under normal conditions. The NH_2_-eGFP fusion protein displayed a diffused and speckled nuclear distribution (Figure 6C, top panels), which was similar to the pattern observed for TcSR62-eGFP. Interestingly, we also noticed that the NH_2_-eGFP protein was also localized in the cytoplasm but in very few parasites (less than 1%, not shown). The deletion mutant COOH-eGFP was also localized to the nucleus and concentrated into speckles in 70% of parasites under normal conditions (Figure 6D, top panels). Remarkably, the remaining parasites presented an exclusively nucleolar localization (not shown), suggesting that under normal conditions TcSR62 might have a nucleolar phase. Then, we tested the localization of these constructs upon ActD treatment. The fusion proteins TcSR62-eGFP and COOH-eGFP were accumulated into the nucleolus in 85% and 93% of parasites respectively (Figure 6B and 6D, bottom panels); however, the localization of the NH_2_-eGFP protein remained mostly unchanged in 95% of parasites (Figure 6C, bottom panels). Control experiments expressing eGFP alone presented a diffuse signal throughout the entire parasite in both untreated and ActD-treated parasites (Figure S3). The graphic in panel (E) is a quantification of the experiments showed in panels (B), (C) and (D).

Altogether, these results suggest that both the NH_2_- and COOH-regions of TcSR62 are able to promote its nuclear localization. However, the nucleolar localization determinant(s) seems to be present exclusively in the COOH terminal region.

### Nucleolar Accumulation of Poly(A)+ RNA is induced under ActD treatment

**Figure 7:**
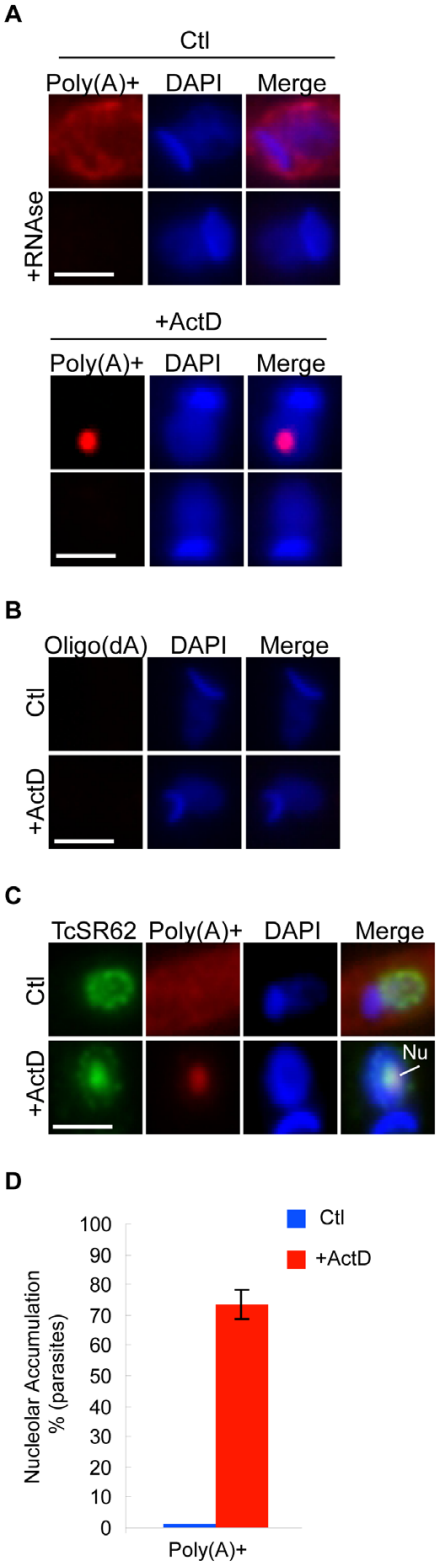
Poly(A)+ RNA is accumulated into the nucleolus in response to ActD treatment. (A) Poly(A)+ was detected by FISH using a Cy3-labelled oligo(dT)30 probe in parasites untreated or incubated with ActD for 24 h. In addition, cells were pre-treated with RNAse A before performing FISH (bottom panels). (B) RNA FISH using a Cy3-labelled oligo(dA)30 probe in parasites untreated or incubated with ActD for 24 h. (C) Immunofluorescence against TcSR62 (green) coupled to FISH using a Cy3-labelled oligo(dT)30 probe in parasites untreated or incubated with ActD for 24 h. Nuclei were counterstained with DAPI (blue). Representative nuclei are shown. Nu: nucleolus. Size bars represent 2 µm. The graphic in panel (D) is a quantitative analysis of poly(A)+ behaviour. Results are expressed as mean +/− SD from at least three independent experiments.

Since some RBPs involved in mRNA metabolism were relocalized to the nucleolus in response to ActD treatment, we wondered whether mRNAs could also be mobilized to the nucleolus under such condition. To address this issue, we carried out RNA-FISH using a 30 mer-oligo(dT) probe against the polyA tail sequence characteristic of mRNAs. Figure 7A and S5 shows that in untreated parasites (left panels) poly(A)+ RNA was detected predominantly in the cytoplasm, with less intense staining in the nucleoplasm. After subjecting parasites to ActD treatment for 24 h, the cytoplasmic poly(A)+ RNA signal decreased significantly, while the nuclear signal concentrated into the nucleolus in 73% of the parasites (Figure 7A right panels and S5, see Figure 7D for a quantitative analysis). As controls, we performed i) RNAse A digestion before the hybridization step (Figure 7A bottom panels and S5), and ii) RNA-FISH using an 30 mer-oligo(dA) probe (Figure 7B). In both control experiments, the fluorescence remaining after the hybridization was negligible, thus confirming that we detected RNA, and that the probe was specific. Finally, we confirmed the nucleolar accumulation of poly(A)+ RNA by colocalizing poly(A)+ with TcSR62 (Figure 7C), thus confirming that RNA poly(A)+ is indeed relocated to the nucleolus under this stress condition.

### Severe heat shock reversibly promotes nucleolar relocalization of both TcSR62 and TcPTB2 but not of TcPABP1

**Figure 8:**
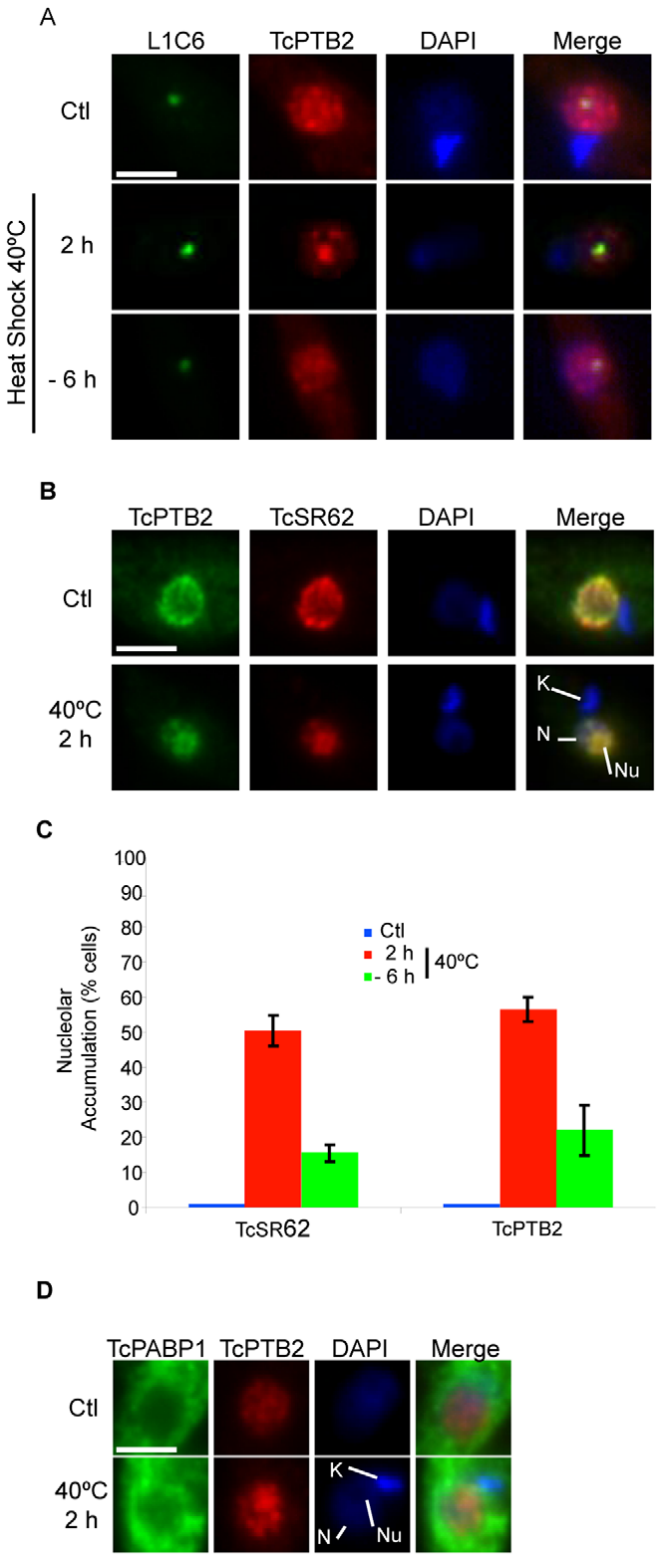
Severe heat shock could reversible promote nucleolar localization of both TcSR62 and TcPTB2 but not TcPABP1. (A) Immunofluorescence images of double labelling for nucleolar marker L1C6 (green) and TcPTB2 (red), under normal conditions, 2 h at 40°C or 6 h of recovery at 28°C. (B) TcPTB2 (green) and TcSR62 (red) in untreated cells or cells incubated at 40°C for 2 h. Nuclei were counterstained with DAPI (blue). The graphic in panel (C) shows the percentage of cells showing nucleolar localization for TcSR62 and TcPTB2 in control (blue bars), at 40°C 2 h (red bars) and recovered at 28°C for 6 h (green). The results are expressed as mean +/− SD from three independent experiments. (D) TcPABP1 (green) and TcPTB2 (red) in untreated cells and cells incubated at 40°C for 2 h. The fourth column on the right is an overlap of TcPTB2, DAPI and each protein evaluated. N: nucleus Nu: nucleolus. K: kinetoplast. Size bars represent 2 µm. Representative nuclei are shown.

As shown before (Figure 1A), nucleolar mobilization of RBPs was only promoted by long-term ActD-treatment, clearly a non-physiological condition. If this kind of response is indeed a physiological one, we thought that it should be an environmental- or cellular-stress that triggers this specific response. It is well known that in higher organisms, heat shock induces an elaborate stress response that involves different signalling pathways [57], which, among other things, shuts down the transcription of most genes [58], [59]. In epimastigotes, which are normally cultured at 28°C, the classical heat shock response is initiated at 40°C [60]. To verify whether this severe heat shock might promote nucleolar accumulation of RBPs, we analyzed the localization of TcPTB2, TcSR62 and TcPABP1 in parasites subjected to heat shock at 40°C for 2 h. TcPTB2 was partially accumulated in the nucleolus in 49% of the parasites (Figure 8A, middle panels and Figure 8C). It is noteworthy that rather than a complete nucleolar accumulation of TcPTB2 —as that observed upon ActD treatment—, we observed an evident but partially accumulation of TcPTB2, since speckles were still visible. Then, we asked whether such nucleolar accumulation was also dependent on phosphorylation and an active transport mechanism, as previously shown in parasites subjected to ActD treatment (see Figure 5). As expected, similar results were observed upon heat shock treatment (Figure S6).

Interestingly, after parasites were returned to normal temperature conditions (6 h at 28°C), TcPTB2 resumed its nuclear speckled pattern in 71% of the parasites (Figure 8A, bottom panels and Figure 8C), demonstrating the reversible nature of this response. Similarly, TcSR62 was also partially accumulated into the nucleolus in 48% of the parasites (Figure 8B, bottom panels and Figure 8C). Surprisingly, TcPABP1 remained in the cytoplasm, displaying a more intense signal around the nucleus (Figure 8D, bottom row).

It should be mentioned that most parasites showed a diffused nuclear pattern of the nucleolar marker L1C6, even to a greater extent than in parasites treated with ActD. This fact prevented us to use it to colocalize the analyzed proteins with the nucleolus.

These results show that severe heat shock --an environmental stress that can be faced by the parasite insect forms-- also promotes nucleolar accumulation of TcSR62 and TcPTB2.

Interestingly, the absence of nucleolar accumulation of TcPABP1 suggests that although transcription could also be inhibited by severe heat shock, the pathway activated may be somehow different or, alternatively, this harsh conditions might inactive some transporters needed by cytoplasmic RBPs to shuttle into the nucleus.

## Discussion

Recently, the resolution of human and *Arabidopsis* nucleolar proteomes have unexpectedly shown the presence of proteins that are known to be involved in several steps of mRNA metabolism such as export, splicing and quality control [14], [17]. The nucleolar localization of these factors supports a growing line of evidence, which argues in favour of a yet undefined role of the nucleolus in mRNA metabolism. In this frame, we present evidence that *T. cruzi* RBPs involved in mRNA metabolism, such as splicing (TcSR62, TcPTB2) or translation (TcPABP1), in addition to poly(A)+ RNA, are accumulated in the nucleolus in response to ActD treatment, supporting the notion that the additional roles of the nucleolus could have been acquired early in the evolution of the eukaryotes, since trypanosomes are an early divergent group of this lineage.

Surprisingly, we have recently found that the mechanism/pathway behind the stress-induced nucleolar accumulation of RBPs is absent in *T. brucei*, a close relative of *T. cruzi* (Názer et al., in preparation), thus suggesting a different degree of conservation during the evolution of the trypanosomatid lineage.

In this report, we showed that nucleolar accumulation of TcSR62, TcPTB2 and TcPABP1 requires an energy-dependent mechanism since nucleolar localization of these proteins in response to ActD treatment was abolished when parasites were incubated either at 4°C or in the presence of metabolic inhibitors (Figure 4). These results are in agreement with recently published results that show that nucleolar localization of p53 in response to proteosomal inhibition occurs in an ATP-dependent manner [61].

We also showed that the nucleolar localization of the RBPs analyzed is also modulated by the activity of kinases but not phosphatases. In other cell systems such as mammals and plants, it has been shown that phosphorylation may modulate the cellular localization of RBPs such as PTB, PABP and SR proteins, [46]–[48], and that inhibition of the phosphorylation/dephosphorylation cycle promotes the redistribution of specific SR proteins in the nucleus [47], [56], [62]. More recently, it has been demonstrated that phosphorylation inhibition induces nucleolar retention of the SR protein atRSZp22 in *Arabidopsis* [62]. In *T. cruzi*, our kinase inhibition results (Figure 5 and S6) suggest that phosphorylation also has an important role in the modulation of the relocalization of specific RBPs when these parasites are under certain stress conditions.

Protein sequence elements for targeting to subnuclear compartments are quite diverse in eukaryotes. For example, in HeLa and *Arabidopsis* cells, both the RRM and RS domains are able to target SR and SR-related proteins to the nucleus. However, the determinants to allow their nuclear speckled distribution are mainly localized into the RS domain [62]–[64]. Here, we showed that GFP-tagged deletion constructs of TcSR62 encompassing either the three RRMs or the COOH-terminal domain were localized in nuclear speckles under normal growth conditions. Interestingly, the last construct was also targeted to the nucleolus in about 30% of the parasites, thus suggesting that TcSR62 could have a nucleolar phase under normal conditions. When parasites were subjected to ActD treatment, only the deletion construct containing the COOH-terminal domain was localized into the nucleolus (Figure 6D). Interestingly, bioinformatic sequence analysis of TcSR62 showed the presence of a basic amino acid-rich region bearing a putative bipartite nuclear localization signal (NLS) in this domain (aa 365–380, predicted using Prosite with a score of 4). This result is in agreement with a previous study in *T. cruzi* showing the involvement of a bipartite NLS as a targeting signal to the nucleolus for the metacyclic-specific protein Met-III [22]. Taken together, our results suggest that both the NH_2_- and the COOH-terminal domains bear sequence elements to target TcSR62 to nuclear speckles, whereas only the carboxy terminal region contains the targeting region (maybe a NLS) to the nucleolus which might play a role in modulating the shuttling between speckles and the nucleolus. However, additional signals, such as phosphorylation (see above), may be needed to promote the transport of this protein to the nucleolus under stress conditions.

One point of concern raised by our results was that nucleolar relocalization of RBPs and poly(A)+ RNA were seen only after 24 h of ActD treatment, even though BrUTP incorporation experiments showed that transcription was abolished after 2 h of ActD treatment (not shown). However, a time-course experiment (Figure S1) provided evidence that the parasite started to accumulate proteins into the nucleolus after 9 h, although the response was more evident at 24 h, when about 86% of the parasites displayed a nucleolar localization of the proteins assayed. A period of 9 h is still a long delay for an effective physiological response to take place. So, we thought to find out another condition that could induce the same response but faster. Interestingly, severe heat shock induced the same type of response after only 2 h at 40°C. So, we thought that the late response induced by ActD is probably due either to the activation of an alternative and slower pathway leading to the same type of response or to an ActD side effect that prevents an early response. Anyway, ActD was a useful drug to first discover and then characterize a novel behaviour in *T. cruzi*.

We thought that the nucleolar relocalization of RBPs and poly(A)+ RNA in *T. cruzi* is indeed part of a specific response induced by transcription inhibition because i) it affects only a particular subset of RBPs; ii) it is activated by transcription inhibitors (and also by severe heat shock) and not by many other stresses assayed (see Figure 1A); iii) neither trans-splicing nor translation inhibitors promote such response; iv) it depends on an active transport mechanism and is modulated by the phosphorylation status of the cell; and v) it is a reversible response as shown by the severe heat shock experiment (Figure 8). Taken together, all these characteristics are compatible with a physiological response and not simply with an aggregate of proteins and RNAs.

What is the biological meaning of RBPs nucleolar relocalization in response to transcription inhibition? At present, we have two non-mutually exclusive working hypotheses. One is that under normal conditions, these proteins have a nucleolar phase which could be evidenced under transcription inhibition as it has been reported for several RBPs in other cell systems [16], [62], [65]. Alternatively, and as previously suggested [12], [13], [22], [65], the nucleolus could act both as a stress sensor and a stress-response mediator by sequestering gene expression regulators in order to remodel the gene expression pattern. Interestingly, we also detected the presence of poly(A)+ RNA (presumably mRNA) into the nucleolus in parasites subjected to transcription inhibition (Figure 7). The fact that the FISH assays were performed 24 h after transcription inhibition, when most mRNA is expected to be degraded, suggests that the nucleolus could behave also as a degradation resistant focus for mRNAs under particular stress conditions. Experiments to further characterize the behaviour of mRNAs under such conditions are under way.

## Materials and Methods

### Trypanosomes

*T. cruzi* CL Brener epimastigotes were cultured in BHT medium containing brain heart infusion, 0.3% tryptose, 0.002% bovine hemin and 10% heat-inactivated fetal calf serum (BHT 10%) at 28°C. Parasite cultures were taken in a late logarithmic growth phase at a cell density of 2.5–3.5×10^7^ parasites ml-1.

### Reagents and Treatments

Transcription inhibition was induced incubating *T. cruzi* parasites with Actinomycin D 50 µg/ml (Sigma) for 24 h. cycloheximide 100 µg/ml, okadaic acid 100 µM, staurosporine 20 µM, sodium azide 10 mM and 2-Deoxy-Glucose 10 mM were purchased from Sigma. Phleomycin was used at a final concentration of 100 µg/ml (InvivoGen), chloroquine at 1 mg/ml and sinefungin at 10 µg/ml. For heat shock experiments, log-phase epimastigotes were incubated at 37°C for 24 h, or at 40°C for 2 h in a water bath. Acid pH stress was induced incubating parasites in BHT pH 5.5 for 24 h.

### Protein Extract

For total extract preparation parasites were resuspended in lysis buffer (10 mM Tris-HCl (pH 7.6), 150 mM NaCl, 50 µM E64 (trans-epoxy succinyl amido 4-guanidino), 1 mM phenylmethylsulfonyl fluoride and 0.5% Nonidet P-40 and incubated on ice for 15 min and then mixed with one volume of reducing cracking buffer 2X.

### Western Blotting

Total protein extracts from mock-treated epimastigotes or epimastigotes exposed to ActD were separated by SDS-PAGE (10%) and blotted on Immobilon-P filters (Millipore). The membranes were blocked with TBS, 3% non-fatted milk, for 60 minutes at room temperature (RT). Primary and secondary antibodies were diluted in blocking buffer plus 0.05% Tween20 and incubated for 1 h 30 minutes and 1 h, respectively, at RT. Primary antibodies used were polyclonal rabbit anti-TcSR62 (1∶1000) and polyclonal rabbit anti-TcGDH (1∶4000). The secondary antibody used was horseradish peroxidase-conjugated goat anti-rabbit IgG (1∶4000) and was developed with the Supersignal® West Pico Chemiluminescent Substrate (Pierce) according to the manufacturer's instructions.

### Immunofluorescence

Trypanosomes were centrifuged from log phase cultures for 2 minutes at 2000 g, washed in PBS twice, allowed to settle on poly-L-lysine-coated slides and fixed in paraformaldehyde (PFA) 4% in PBS at RT for 10 minutes. After two brief washes in PBS at RT, fixed cells were incubated at RT with 25 mM NH_4_Cl in PBS for 10 minutes. Cells were washed twice with PBS, permeabilized and blocked with 0.5% saponin, 1% Bovine serum albumin, 2% goat normal serum in PBS for 60 minutes at RT. After blocking, cells were first incubated with the primary antibody (diluted in 0.1% saponin and 1% BSA in PBS) for 60 minutes and then washed 3 times with PBS. Afterwards, slides were incubated with secondary antibodies (diluted in 0.1% saponin and 1% BSA in PBS) for 60 minutes, washed three times with PBS and once in MQ water. Primary antibodies were monoclonal L1C6 (1∶200), polyclonal anti-TcSR62 (1∶1000), polyclonal anti-TcPABP1 (1∶1000), polyclonal anti-TcPTB2 (1∶1000), polyclonal anti-TcRNP38 (1∶1000), polyclonal anti-TbLA (1∶200), polyclonal anti-TcHSP70 (1∶1000), polyclonal anti-TcSF3b155 (1∶500), and polyclonal anti-TcFip1 (1∶500).

Secondaries goat anti-rabbit or anti-mouse antibodies AlexaFluor 488 or AlexaFluor 594 (Molecular Probes) were used at 1∶1000 dilutions. Finally, cells were mounted in 1 µg/ml DAPI prepared in Fluorsave (Calbiochem). Analysis of subcellular localization was performed in a Nikon Eclipse E600 microscope coupled to a SPOT RT colour camera (Diagnostic Instruments). Merged images were obtained by superimposing the indicated images files in SPOT Software 4.0.9 (Diagnostic Instruments).

### GFP fusion constructs

Full length TcSR62 and its derivative deletions were amplified by PCR using the primers listed below and cloned into the BamHI site of pTEX-eGFP kindly provided by Dr. J.M. Kelly [66].

TcSR62

Fv_TcSR62_BamHI: cgggatccATGtcgtacacgattcagg.

Rv_TcSR62_NoSTOP_BamHI: cgggatccCGgtagcgacggcgcggt.

NH2

Fv_TcSR62_BamHI: cgggatccATGtcgtacacgattcagg.

Rv_TcSR62_NH2_NOSTOP_BamHI: cgggatccCGgttgcggcaatacggct.

COOH

Fv_TcSR62_COOH_BamHI: cgggatccATGgacaatcgccgcaac.

Rv_TcSR62_NoSTOP_BamHI: cgggatccCGgtagcgacggcgcggt.

### Parasite Transfections

Transfections were carried out with a BTX 600 electroporator in a 2-mm gap cuvette. A total of 150×10^6^ parasites were harvested and washed twice with BHT medium, resuspended in 0.35 ml of BHT with 50 µg of supercoiled plasmid DNA. Electroporation settings were: 1400 microfarads, 335 V, and 24 Ω. Parasites were recovered in 4 ml of BHT supplemented with 10% fetal calf serum (Natocor) and 36 h later geneticin (Sigma) was added at a final concentration of 500 µg/ml.

### Fluorescence *in situ* hybridization

For detection of total poly(A)+ RNA by FISH, parasites were harvested, allowed to adhere to poly-lysine-coated microscope slides, fixed with 4% PFA in PBS at RT for 10 minutes, followed by a 10-min incubation with 25 mM NH_4_Cl. Fixed parasites were permeabilized and blocked for 1 h in 0.5% saponin (Sigma), 2% BSA (Blocking Buffer), followed by 2-h prehybridization at RT in 2% BSA, 5× Denhart, 4× SSC, 5% dextran sulphate, 35% deionized formamide (Sigma), 0.5 µg/µl yeast tRNA (Sigma) and 10 U/ml RNAsin (Promega) (Hybridization Solution). Hybridization was performed overnight at 28°C in a humid chamber either in the presence of 1 ng/µl Cy3-conjugated oligo(dT)30 or Cy3-conjugated oligo(dA)30 in Hybridization Solution. Slides were washed twice in 4× SSC at RT. Slides were mounted in 1 µg/ml DAPI prepared in Fluorsave (Calbiochem). RNAse A pretreatment was performed at 37°C for 30 minutes before hybridization.

## Supporting Information

Figure S1 **Time-course behaviour of TcSR62 under transcription inhibition.** Epimastigote parasites were subjected to transcription inhibition with ActD and analysed by immunofluorescence against TcSR62 (green) at the indicated time points. The localization of TcSR62 in few bright speckles (3–9 h) and in the nucleolus (24 h) is indicated by white arrows. Nuclei were counterstained with DAPI (blue). Portion of representative field sections are shown. Size bar represents 2 µm.
(TIF)

Figure S2 **Nucleolar accumulation of TcFLIP1 and TcSF3b155 under transcription inhibition.** Epimastigote parasites were subjected to transcription inhibition with ActD during 24 h. Immunofluorescence using specific antibodies against (A) TcFLIP1 (red) or (B) SF3b155 (red) was carried out. In both cases, each protein was colocalized with TcSR62 (green). The third column on the right is an overlap of each protein and TcSR62. Green and red pixels overlapped in the digital images yield yellow signals. Portion of representative nuclei are shown. Size bar represents 2 µm.
(TIF)

Figure S3 **Localization of eGFP under transcription inhibition.** Epimastigote parasites expressing eGFP alone in the vector pTEX were subjected to transcription inhibition with ActD during 24 h. Nuclei were counterstained with DAPI (blue). The third column on the right is an overlap of eGFP and DAPI. Representative parasites are shown. Size bar represents 2 µm.
(TIF)

Figure S4 **Effects of ActD treatment on the localization of TcPABP1, TcRNP38 and TcHSP70 showing whole parasites.** Immunofluorescence images of the corresponding protein in ActD-treated and untreated epimastigotes. (A)TcPABP1 (in green) was colocalized with the nucleolar marker L1C6 (red). Nuclei were counterstained with DAPI (blue). The fourth column on the right is an overlap of each protein, L1C6 and DNA staining. Green and red pixels overlapped in the digital images yielded yellow signals. Immunofluorescence images for (B) TcRNP38 and (C) TcHsp70. Each protein is shown in green. Nuclei were counterstained with DAPI (blue). The third column on the right is an overlap of each protein and DNA stain. Size bars represent 2 µm. Representative parasites are shown.
(TIF)

Figure S5 **Poly(A)+ RNA is accumulated into the nucleolus in response to ActD treatment.** Poly(A)+ RNA was detected by FISH using a Cy3-labelled oligo(dT)30 probe in parasites untreated or incubated with ActD for 24 h. In addition, cells were pre-treated with RNAse A before performing FISH. Nuclei were counterstained with DAPI (blue). Representative field sections are shown. Size bars represent 2 µm.
(TIF)

Figure S6 **Nucleolar accumulation of TcSR62 induced by severe heat shock depends on an active mechanism modulated by phosphorylation.** Epimastigotes were incubated with either sodium Azide (Az) and 2-Deoxy-Glucose (2De) or Staurosporine (Stau) for 16 h. Then, parasites were incubated at 40°C for 2 h. Immunofluorescences were performed against TcSR62 (green). Parasite culture at 40°C is shown as control. Nuclei were counterstained with DAPI (blue). The third column on the right is an overlap of the TcSR62 and DAPI. Size bar represents 2 µm. Representative nuclei are shown.
(TIF)
