# Supplementary figures and images for: Nucleolar Localization of RNA Binding Proteins Induced by Actinomycin D and Heat Shock in Trypanosoma cruzi
Source: PLoS One. 2011 May 24;6(5):e19920. doi: 10.1371/journal.pone.0019920 (PMC3101214; doi:10.1371/journal.pone.0019920)

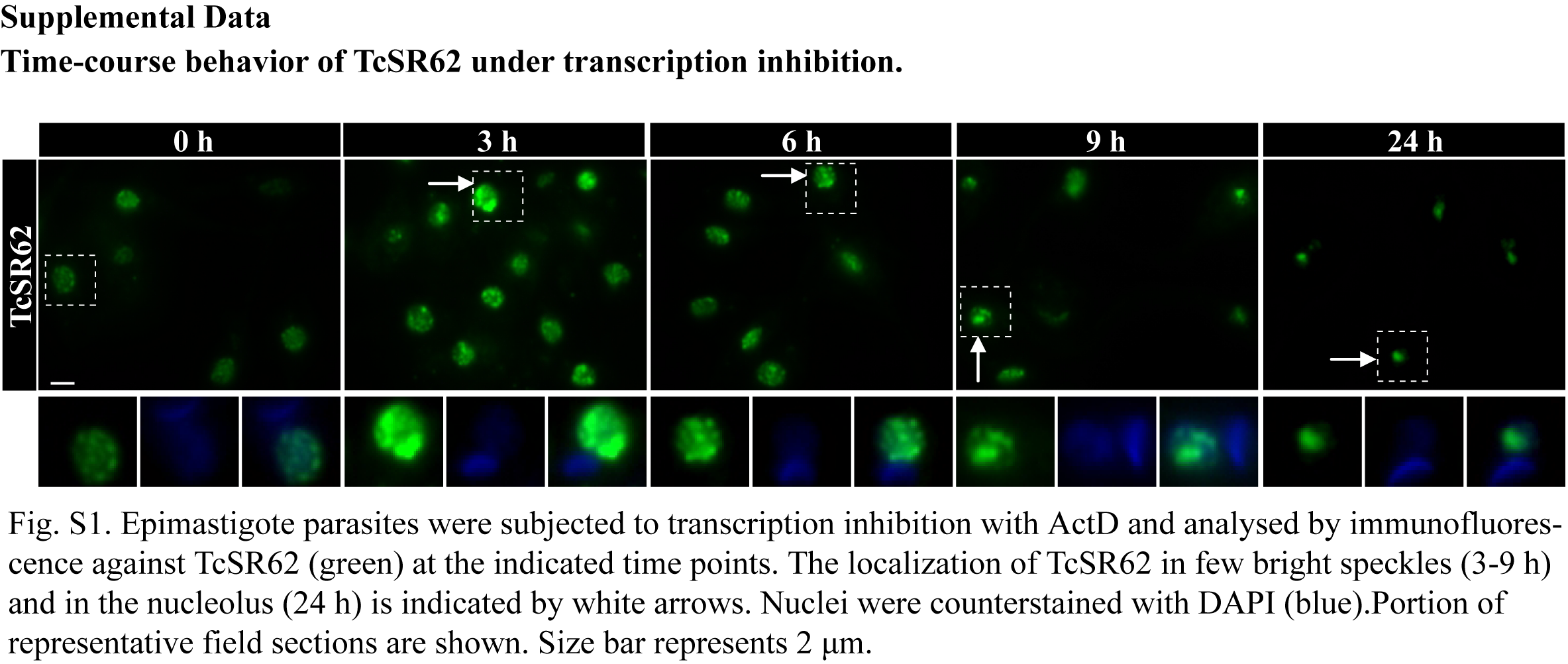

Supplement: Figure S1 — Time-course behaviour of TcSR62 under transcription inhibition. Epimastigote parasites were subjected to transcription inhibition with ActD and analysed by immunofluorescence against TcSR62 (green) at the indicated time points. The localization of TcSR62 in few bright speckles (3–9 h) and in the nucleolus (24 h) is indicated by white arrows. Nuclei were counterstained with DAPI (blue). Portion of representative field sections are shown. Size bar represents 2 µm. (TIF) [file pone.0019920.s001.tif]

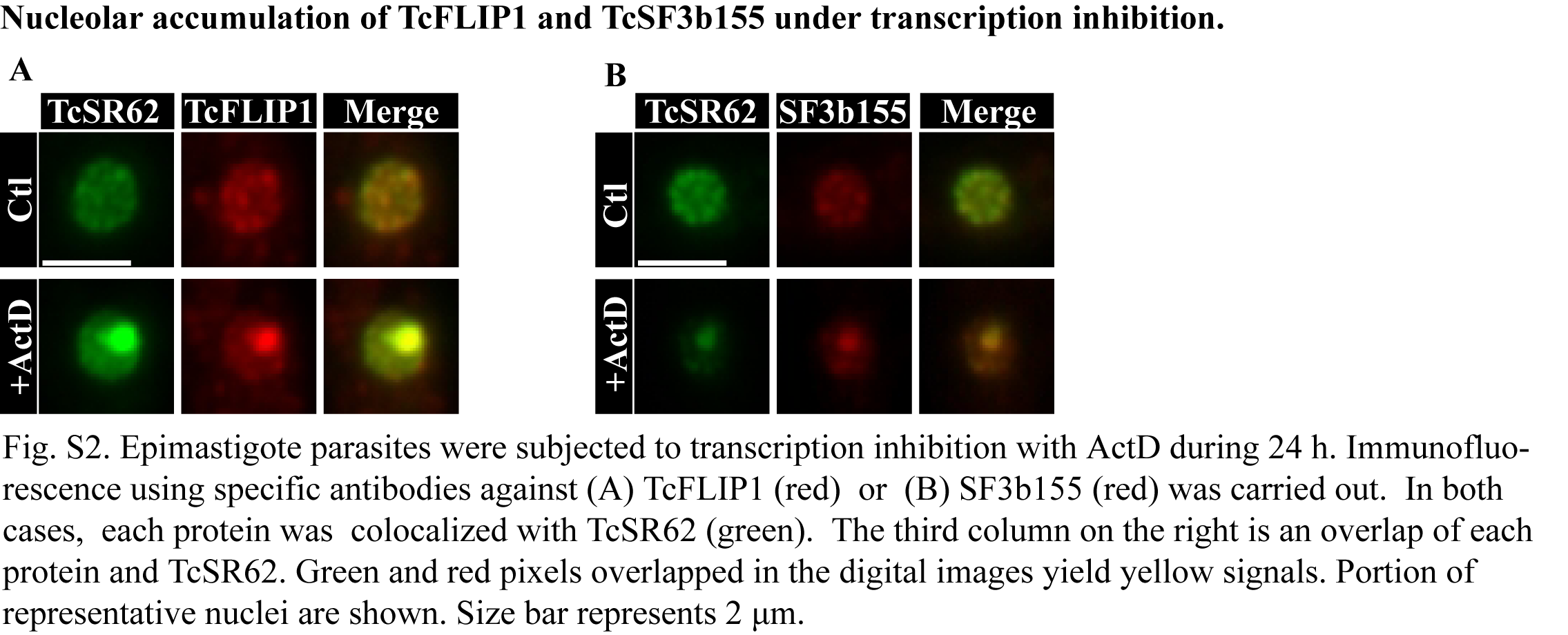

Supplement: Figure S2 — Nucleolar accumulation of TcFLIP1 and TcSF3b155 under transcription inhibition. Epimastigote parasites were subjected to transcription inhibition with ActD during 24 h. Immunofluorescence using specific antibodies against (A) TcFLIP1 (red) or (B) SF3b155 (red) was carried out. In both cases, each protein was colocalized with TcSR62 (green). The third column on the right is an overlap of each protein and TcSR62. Green and red pixels overlapped in the digital images yield yellow signals. Portion of representative nuclei are shown. Size bar represents 2 µm. (TIF) [file pone.0019920.s002.tif]

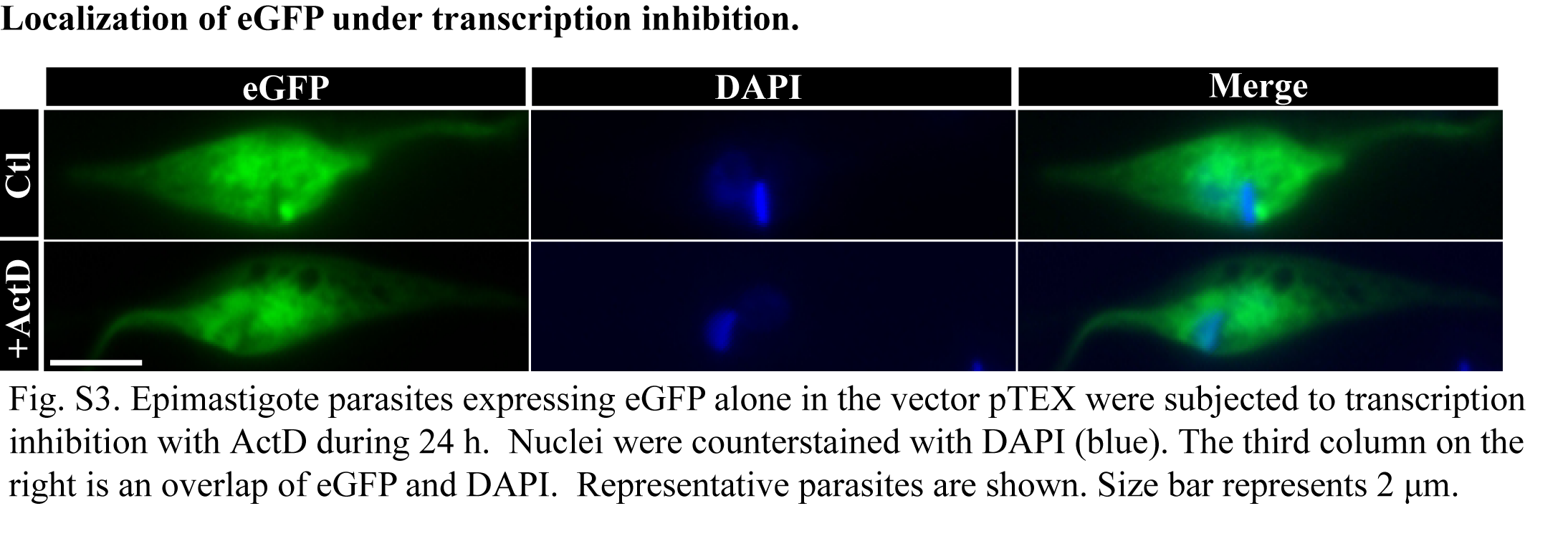

Supplement: Figure S3 — Localization of eGFP under transcription inhibition. Epimastigote parasites expressing eGFP alone in the vector pTEX were subjected to transcription inhibition with ActD during 24 h. Nuclei were counterstained with DAPI (blue). The third column on the right is an overlap of eGFP and DAPI. Representative parasites are shown. Size bar represents 2 µm. (TIF) [file pone.0019920.s003.tif]

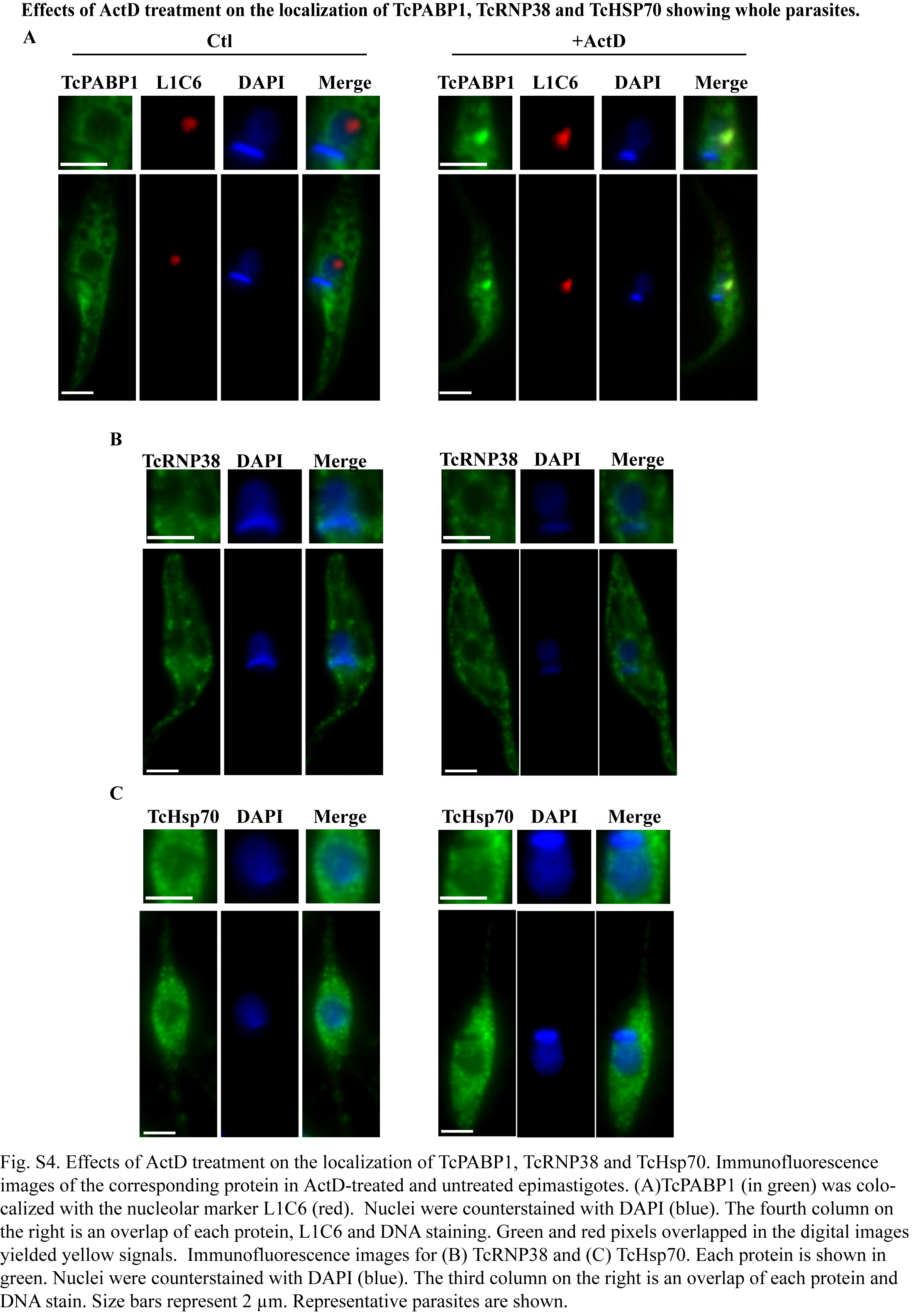

Supplement: Figure S4 — Effects of ActD treatment on the localization of TcPABP1, TcRNP38 and TcHSP70 showing whole parasites. Immunofluorescence images of the corresponding protein in ActD-treated and untreated epimastigotes. (A)TcPABP1 (in green) was colocalized with the nucleolar marker L1C6 (red). Nuclei were counterstained with DAPI (blue). The fourth column on the right is an overlap of each protein, L1C6 and DNA staining. Green and red pixels overlapped in the digital images yielded yellow signals. Immunofluorescence images for (B) TcRNP38 and (C) TcHsp70. Each protein is shown in green. Nuclei were counterstained with DAPI (blue). The third column on the right is an overlap of each protein and DNA stain. Size bars represent 2 µm. Representative parasites are shown. (TIF) [file pone.0019920.s004.tif]

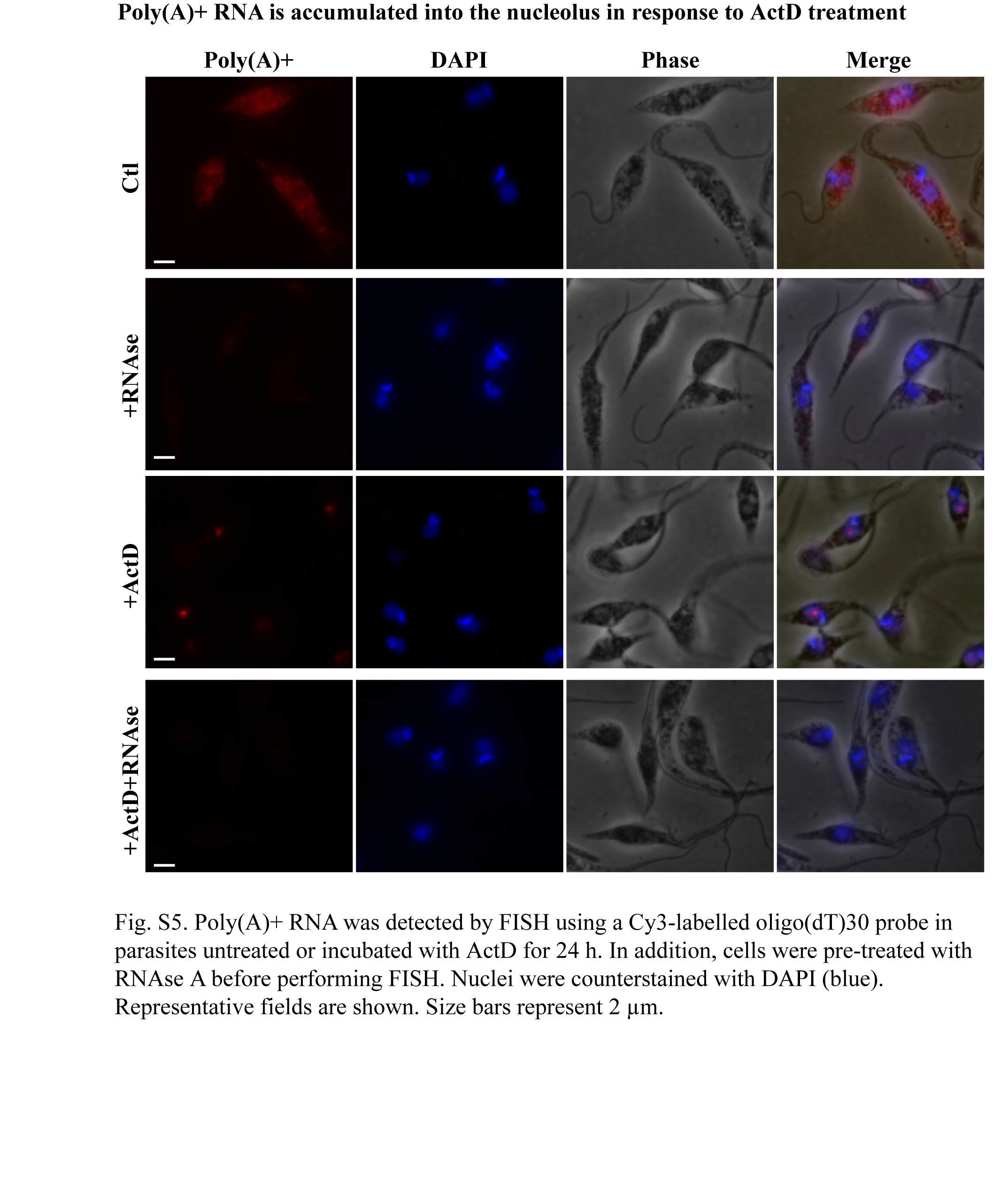

Supplement: Figure S5 — Poly(A)+ RNA is accumulated into the nucleolus in response to ActD treatment. Poly(A)+ RNA was detected by FISH using a Cy3-labelled oligo(dT)30 probe in parasites untreated or incubated with ActD for 24 h. In addition, cells were pre-treated with RNAse A before performing FISH. Nuclei were counterstained with DAPI (blue). Representative field sections are shown. Size bars represent 2 µm. (TIF) [file pone.0019920.s005.tif]

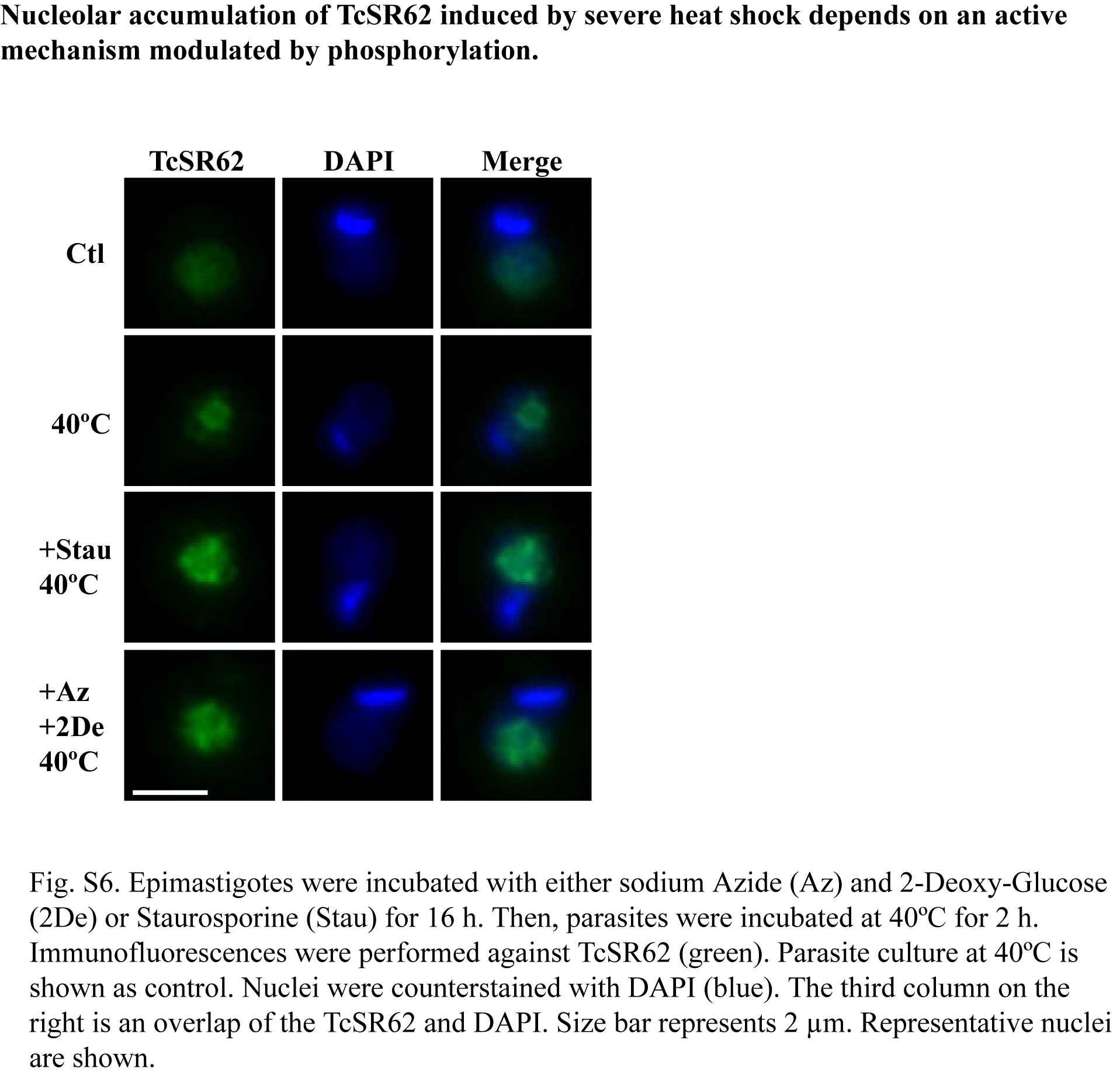

Supplement: Figure S6 — Nucleolar accumulation of TcSR62 induced by severe heat shock depends on an active mechanism modulated by phosphorylation. Epimastigotes were incubated with either sodium Azide (Az) and 2-Deoxy-Glucose (2De) or Staurosporine (Stau) for 16 h. Then, parasites were incubated at 40°C for 2 h. Immunofluorescences were performed against TcSR62 (green). Parasite culture at 40°C is shown as control. Nuclei were counterstained with DAPI (blue). The third column on the right is an overlap of the TcSR62 and DAPI. Size bar represents 2 µm. Representative nuclei are shown. (TIF) [file pone.0019920.s006.tif]
